# Propionic acidemia in Mexico: Clinical and genotypic spectrum

**DOI:** 10.1016/j.ymgmr.2026.101311

**Published:** 2026-04-15

**Authors:** M. Vela-Amieva, M.A. Alcántara-Ortigoza, S. Guillén-López, L. López-Mejía, A. González-del Ángel, M. Reyna-Fabián, L. Fernández-Hernández, Gabriel López-Velázquez, D. Mancera-Hernández, S. Enríquez-Flores, I. Ibarra-González, C. Fernández-Lainez

**Affiliations:** a Laboratorio de Errores Innatos del Metabolismo y Tamiz, Instituto Nacional de Pediatría, Secretaría de Salud, Mexico City 04530, Mexico; b Laboratorio de Biología Molecular, Instituto Nacional de Pediatría, Secretaría de Salud, Mexico City 04530, Mexico; c Laboratorio de Biomoléculas y Salud Infantil, Instituto Nacional de Pediatría, Secretaría de Salud, Mexico City 04530, Mexico; d Unidad de Genética de la Nutrición, Instituto de Investigaciones Biomédicas, UNAM, Mexico City 04530, Mexico

**Keywords:** Inborn errors of metabolism, Newborn screening, Genetic testing, Rare diseases, Organic acidemias, Inherited metabolic diseases

## Abstract

Propionic acidemia (PA) is conditioned by a deficiency of propionyl-CoA carboxylase, whose subunits are coded by the *PCCA* and *PCCB* genes. In the Mexican population, little is known about the clinical presentation of PA and the underlying genotypic *PCCA* and *PCCB* spectrum. PA is not currently assessed in the mandatory Mexican newborn screening (NBS) program. We aimed to characterize the clinical and genotypic spectrum in 51 Mexican patients with PA seen at a national reference center in Mexico. Most of the patients (92.1%) showed an early symptom onset (mean 21 days of life), delayed diagnosis (mean 5.1 months of life), considerable diagnostic odyssey (mean 4.4 months), and a high early mortality rate (66.7%). Feeding difficulties, hyperammonemia, and metabolic acidosis predominated as early PA signs occurring within the first month of life. At the last follow-up, 60% (*N* = 18/30) of patients exhibited profound intellectual and motor impairment. Next-generation sequencing revealed that 46.66% (*N* = 14/30) of cases were *PCCA*-related and 53.3% (*N* = 16/30) were *PCCB*-related. We identified four clinically relevant novel variants in *PCCA* [c.-10_105 + 11del, p.(Gly226Arg), c.1643 + 3 A > G, p.(Ser245*)] and one in *PCCB* [p.(Met463Arg)]. Protein in silico modeling of the *PCCA* p.(Gly226Arg) and *PCCB* p.(Met463Arg) variants predicted structural disturbances supporting their pathogenicity. The c.2041-1G > T [rs1367867218] and c.1309G > A or p.(Gly437Ser) [rs1349202366] variants were the most frequent pathogenic ones in *PCCA* (*N* = 5/28 alleles, 17.9%) and *PCCB* (*N* = 7/32 alleles, 21.9%), respectively. Predominance of neonatal onset, severe neurological sequelae, and high mortality rate emphasize that PA should be considered for inclusion in the Mexican NBS program. Until PA screening is routinely performed at birth in Mexico, efforts are needed to increase pediatricians' awareness of the clinical picture, to support early detection and prompt management.

## 1 Background

Propionic acidemia (PA, OMIM #606054) is an autosomal recessive inborn error of metabolism. It is classified as an organic acidemia and is caused by propionyl-CoA carboxylase (PCC) deficiency. PCC is a mitochondrial enzyme that catalyzes the conversion of propionyl-CoA to methylmalonyl-CoA, which subsequently enters the citric acid cycle as succinyl-CoA. Biotin is an obligate cofactor of PCC [1], [2]. PCC is a heterododecameric 750-kDa complex composed of six propionyl-CoA carboxylase alpha subunits encoded by *PCCA* (13q32.3, MIM*232000) and six propionyl-CoA carboxylase beta subunits encoded by *PCCB* (3q22.3, MIM*232050)[3].

PA is a heterogeneous organic acidemia with onset ranging from the neonatal period to late childhood. Neonatal-onset cases typically present with poor feeding, encephalopathy, metabolic acidosis, hyperammonemia, and cytopenias, whereas later-onset forms may involve multisystem complications affecting growth, neurological function, the cardiovascular system, and other organs [1], [2], [3], [4]. Elevated blood concentrations of propionylcarnitine (C3) and abnormal urinary organic acids, including 3-hydroxypropionate, methylcitrate, tiglylglycine, propionylglycine, and lactic acid, are diagnostic PA biochemical markers. Glycine concentrations in blood are also frequently elevated [1], [3].

Although PA is amenable to detection through newborn screening (NBS), implementation remains inconsistent worldwide because of logistical constraints and the need for specialized analytical platforms and trained personnel [5]. In Latin America, access to expanded NBS based on tandem mass spectrometry is variable, with particularly pronounced disparities in Mexico. The absence of a uniformly implemented national expanded NBS program, combined with the fragmentation of the healthcare system, has resulted in substantial heterogeneity in screening panels, laboratory methodologies, and institutional coverage. Consequently, early identification of PA is not consistently achieved, and the likelihood of timely diagnosis often depends on the healthcare sector and place of birth. This variability contributes to delayed diagnosis, increased risk of metabolic decompensation, and potentially worse clinical outcomes, thereby amplifying the burden of disease and healthcare costs associated with inborn errors of metabolism. National analyses have documented wide inter-institutional differences in the number of disorders screened, ranging from minimal panels to expanded programs, underscoring persistent inequities in access to timely detection and intervention [6], [7], [8].

Acute management of PA focuses on rapid reversal of catabolism by ceasing or restricting protein intake, administering intravenous high-carbohydrate fluids, and, when indicated, using ammonia scavengers and hemodialysis. l-carnitine and antibiotics such as metronidazole are also commonly used as adjunctive therapies. Carnitine conjugates with propionic acid, facilitating its detoxification and transport out of the mitochondria, thereby enabling urinary excretion as propionylcarnitine, whereas metronidazole primarily decreases intestinal propionate production by targeting propionate-producing gut bacteria. Long-term management of PA includes a protein-restricted diet, l-carnitine supplementation, periodic use of gut microbiota-targeting antibiotics, and careful biochemical monitoring to prevent metabolic decompensation. In severe cases, orthotopic liver transplantation may be considered [1], [9], [10]. Other emerging therapeutic options are arising, including gene therapy and mRNA-based strategies aimed at restoring enzyme activity, although they are not yet clinically available [11], [12].

The molecular spectrum of PA has been primarily characterized in European, North American, Chinese, and Middle Eastern populations [1]. Its description in Latin American populations remains scarce, with only isolated case reports or case series from countries such as Brazil, Chile, and Argentina [13], [14], [15]. Understanding the genetic landscape of PA in underrepresented populations is crucial for improving diagnosis, providing genetic counseling, and developing potential therapeutic strategies.

Therefore, the aim of this study was to characterize the clinical, biochemical, and molecular features of patients with PA in a Mexican cohort, and to explore potential genotype-phenotype correlations associated with disease severity and outcomes. By describing the diagnostic trajectories, clinical presentation, and molecular variants identified in this population, we sought to identify factors associated with delayed diagnosis and adverse clinical evolution within a national tertiary referral center for inherited metabolic disorders in Mexico. In addition, the findings may also be applicable to other low- and middle-income countries or healthcare systems with similar structural fragmentation and resource constraints, where access to expanded NBS remains heterogeneous and delayed diagnosis continues to impact clinical outcomes.

## 2 Methods

### 2.1 Study design

An observational, descriptive, retrospective-prospective (historical) cohort study was conducted. The retrospective data were collected from 1995 onwards. The prospective component was data obtained from patients still in follow-up (clinical status).

### 2.2 Studied population

The studied population consisted of Mexican patients with biochemical confirmation of PA, drawn from the inborn errors of metabolism cohort of the National Institute of Pediatrics, from 1995 to June 2025. A confirmed PA case was one that met the biochemical criteria of: 1) increased C3 measured by tandem mass spectrometry (MS/MS); and 2) high urine levels of 3-OH-propionic acid, methylcitric acid, propionylglycine, and tiglylglycine determined by gas chromatography coupled to mass spectrometry (GC/MS), as previously reported [16].

### 2.3 *PCCA* and *PCCB* gene sequencing

Genomic DNA was isolated from peripheral blood leukocytes or buccal epithelial cells using a commercially available silica membrane-based extraction kit (QIAamp DNA Blood Mini Kit, Hilden, Germany), following the manufacturer's instructions.

In order to establish the PA-related genotypes of the patients, clinical exome sequencing was selected. This was primarily because exome analysis was available for the enrolled patients within our institutional diagnostic and research framework at the time of recruitment.

Clinical exome sequencing was performed for targeted bioinformatic genetic analysis of *PCCA* (NM_000282.4) and *PCCB* (NM_000532.5). Library preparation was performed using a Flex for Enrichment kit, and target enrichment of coding regions was applied using the TruSight One Expanded panel. Sequencing was performed on the Illumina platform. To that end, paired-end reads (2 × 150 bp) were generated using high-throughput capabilities. FastQC (version 0.12.0, https://www.bioinformatics.babraham.ac.uk/projects) was used to assess the quality of raw sequencing data. Burrows–Wheeler transform was used to align the reads to the GRCh38 reference genome [17]. The Genome Analysis Toolkit (GATK4) [18] and the SnpEff program [19] were used for variant calling and variant annotation, respectively. The Integrative Genomics Viewer (IGV) was used for visual review of the identified variants. For analysis of copy number variation (CNV) in the *PCCA* and *PCCB* genes, the Franklin Genoox platform (https://franklin.genoox.com/clinical-db/home, accessed November 14, 2024) was utilized. The genotypic criterion for identifying a PA case as *PCCA*- or *PCCB*-related was the demonstration of a biallelic genotype involving pathogenic variants, likely pathogenic variants, or variants of unknown significance (VUS) in *PCCA* or *PCCB*.

From the original clinical exome laboratory reports, an independent reassessment of variant classification was performed. This integrated clinical, biochemical, and familial information, along with detailed phenotypic correlations, segregation data, and comparisons with other patients presenting similar genotype–phenotype associations. This was performed according to the guidelines established by the American College of Medical Genetics and Genomics (ACMG) and the Association for Molecular Pathology (AMP) for variant interpretation [20].

The distribution of biallelic loss-of-function (LOF) genotypes was compared between PCCA-related and PCCB-related patients. LOF variants were defined as nonsense, frameshift, and canonical splice-site variants.

### 2.4 Data collection

For the retrospective analysis, data were collected from medical records and our laboratory database. Clinical outcomes were recorded only in patients with molecular confirmation of PA, and these data were used to explore differences in patients affected with variants in *PCCA* or *PCCB*.

### 2.5 Symptom spectrum recording

The symptom spectra were recorded as HPO terms [21].

### 2.6 Diagnostic odyssey

The diagnostic odyssey was defined as the time elapsed from the onset of symptoms to the establishment of the biochemical or molecular diagnosis.

### 2.7 Nutritional management

Nutritional management was implemented in accordance with the international guidelines of the Southeast Regional Genetics Network [22]. Dietary treatment consisted of controlled protein restriction to reduce the accumulation of propionic acid derived from propiogenic amino acids. Individualized protein and energy prescriptions were calculated to ensure adequate intake to support normal growth and prevent endogenous catabolism [22].

The use of specialized medical formulas restricted in propiogenic amino acids, as recommended by the SERN guidelines, was reserved only for patients who were unable to achieve their age-specific recommended dietary allowance (RDA) for natural protein intake, since their use in propionic acidemia remains controversial due to the potential risk of branched-chain amino acid imbalance [22], [23]. Patients underwent regular biochemical monitoring, including plasma amino acid profiling, to guide dietary adjustments and optimize metabolic control.

### 2.8 Outcome measures

The primary outcome measures included survival status, loss to follow-up, neurological outcomes, and functional outcomes. Mortality data were obtained from death certificates, medical records, or direct reports from the patients' parents.

In patients under 6 years of age, neurodevelopmental delay was evaluated using the Denver Developmental Screening Test [24]. This standardized tool assesses developmental progress in four domains: personal-social, fine motor-adaptive, language, and gross motor skills. For patients aged 6 years and older, we used the Patel classification, which categorizes patients by the severity of cognitive and motor impairments. Patients were stratified into four clinical severity groups: asymptomatic, alive with moderate intellectual and motor disability, alive with profound intellectual and motor disability, and deceased with profound intellectual and motor disability[25].

### 2.9 In silico protein modeling predictions

To predict the potential deleterious effects of the newly identified missense *PCCA* and *PCCB* variants, the quaternary structure of the wild-type (Wt) PCAA-PCCB protein complex was compared with that formed by the proteins resulting from the novel variants. Molecular modeling was performed using the crystallographic structure of the PCCA–PCCB protein complex (Protein Data Bank (PDB) code 8XL5, https://www.rcsb.org/, accessed May 14, 2025). All models and figures were constructed with PyMOL software (PyMOL Molecular Graphics System, version 2.0, Schrödinger, LLC, New York, NY, USA) [26]. In the first step, we modeled the locations of amino acid residues in the novel missense variants and gathered details on their near contacts within a 10-Å^2^ area. The solvent-accessible surface area (SASA) was calculated for the amino acid residues of the new variants using the GETAREA tool [27]. The protein's degree of solvent exposure was calculated using the B′-factor with the BANΔIT tool [28]. In silico mutagenesis was performed using PyMOL to predict how the substituted residues would affect neighboring residues.

### 2.10 Statistical analysis

Clinical data were collected by reviewing medical records. All statistical analyses were performed with GraphPad Prism software (version 10.4.2, San Diego, CA, USA). For descriptive statistics, the data distribution was assessed using the Shapiro–Wilk test. For parametric data, one-way ANOVA, mixed-effects analysis with the Geiser–Greenhouse correction, and Dunnett's multiple comparison tests were conducted. Results with a parametric distribution are presented as means ± standard deviations (SDs). The Mann–Whitney *U* test was used for nonparametric data, followed by Dunn's multiple-comparison test, with results presented as medians [Q1–Q3]. Fisher's exact test was used to assess significant differences in proportions for qualitative variables. Confidence intervals for the proportions of clinical symptoms were calculated using the Clopper-Pearson method. Kaplan–Meier survival curves were generated for the overall cohort and stratified by the affected gene (PCCA and PCCB). To evaluate differences in the survival between PCCA-related and PCCB-related patients, Mantel-Cox test was performed.

## 3 Results

Fifty-one PA patients were identified from 45 families, including nine families with affected sibling pairs (9/45, 20%). These families came from 20 of the 32 Mexican states (62.5%), indicating that our study population had a wide national distribution. The genetic study was performed only in 30/51 unrelated patients (58.8%). In all of them, the presence of two variants in PCCA or PCCB was observed.

### 3.1 Perinatal and early postnatal history

All patients were born at term and had normal birth weight, except for one case. Advanced resuscitation at birth was required in only one patient. Five patients (9.8%) required immediate hospitalization upon birth due to respiratory distress. The remaining newborns were discharged home as healthy-term infants. One patient (1/51; 1.9%) underwent an expanded NBS that included C3 determination by MSMS; the remaining patients had the mandatory Mexican NBS, which includes congenital hypothyroidism, congenital adrenal hyperplasia, cystic fibrosis, classical galactosemia, phenylketonuria, and glucose-6-phosphate dehydrogenase deficiency, but not C3 determination.

### 3.2 Clinical picture

**Fig. 1:**
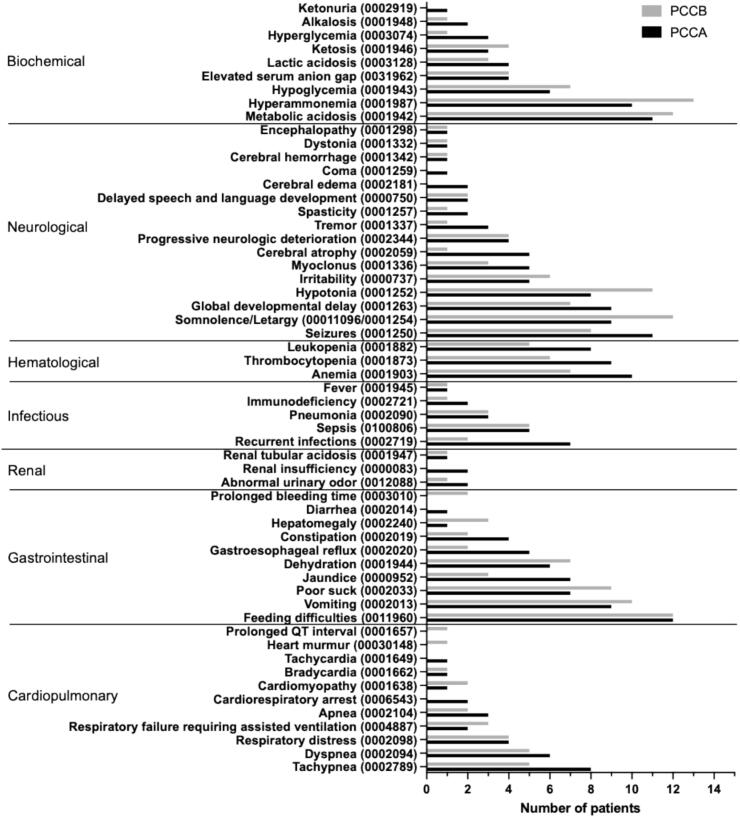
Clinical spectrum of Mexican patients with propionic acidemia. Symptoms were recorded at the time of the initial clinical evaluation in our reference center and were divided by the affected gene (*PCCA* or *PCCB*). The HPO number is shown in parentheses.

The clinical characteristics of Mexican PA patients, categorized by the affected gene (*PCCA* vs. *PCCB*) and presented with their respective HPO codes, are shown in Fig. 1. Overall, the main clinical characteristics of our Mexican PA patients were feeding difficulties (0011960, 80%), hyperammonemia (0001987, 76.7%, mean 408 μmol/L, minimum 48 μmol/L, maximum 1470 μmol/L), metabolic acidosis (0001942, 76.7%), somnolence/lethargy (00011096/0001254, 70%), seizures (0001250, 63.3%), vomiting (0002013, 63.3%), hypotonia (0001252, 63.3%), and global developmental delay (0001263, 53.3%). In this study the symptoms in *PCCB-*related PA patients were more frequent than in *PCCA*-related PA patients (*p* < 0.01). The confidence intervals of clinical symptoms are shown in Supplementary Table S1.

### 3.3 Onset and diagnostic Odyssey

Most PA patients (47/51, 92.1%) had early symptom onset (median 1.8 days [1–8 days]), with a median age at diagnosis of 1.9 months [16 days – 5 months]. The diagnostic odyssey, defined as the period from the onset of symptoms to the diagnosis, lasted 1.3 months [12 days – 4 months].

### 3.4 Patient outcomes

**Fig. 2:**
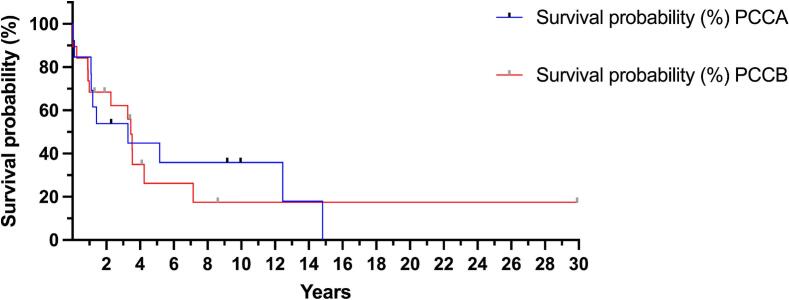
Kaplan Meier survival curves of patients with propionic acidemia by affected gene. Mantel-Cox test revealed no statistical differences between both curves.

At the time of this report, 10 of the PA patients (19.6%) were known to be alive, and seven patients (13.7%) had been lost to medical follow-up. The median current age of the living PA patients was 4.1 years [2–9.5]. Thirty-four of the 51 patients (66.7%) had died, with a median age at death of 17 months [3.5 months – 4 years]. Their main causes of death are shown in Table 3. The Kaplan-Meier survival curve (Fig. 2) shows that PCCA-related and PCCB-related patients had almost 20% probability of survival to age 15 and 30 years, respectively. However, when comparing their survival curves, there was no statistical difference between them.

At the last follow-up, independently of the affected gene, all the patients presented neurological impairment (Table 1). Age-specific recommendations for protein (intact and total) and energy intake, according to the SERN guidelines, are summarized in Supplementary Table S2. The clinical and nutritional evaluation of the patients who are alive is ongoing; thus, further studies are guaranteed. At their last nutritional evaluation, most of the patients were eutrophic.

**Table 1:** Genotypes of Mexican patients with propionic acidemia.

Patient ID	Allele 1	Allele 2	Change protein 1	Change protein 2	ACMG classification allele 1	ACMG classification allele 2	Outcome
PCCA							
PA-13	c.2041-1G > T	c.2041-1G > T	p.(?)	p.(?)	Pathogenic	Pathogenic	Dead at 1.4 years old, with profound intellectual and motor dissability
PA-28	c.2041-1G > T	c.2041-1G > T	p.(?)	p.(?)	Pathogenic	Pathogenic	Dead at 1.2 months, with neurodevelopmental impairment
PA-12	c.2041-1G > T	c.866_867delAG	p.(?)	p.(Glu289Valfs*53)	Pathogenic	Pathogenic	Dead at 14 years old, with profound intellectual and motor dissability
PA-02	c.437 T > C	c.1071G > T	p.(Leu146Pro)	p.(Glu357Asp)	Likely pathogenic	Pathogenic	Dead at 1.5 years old, with profound intellectual and motor dissability
PA-20	c.674G > A	c.1429G > A	p.(Gly225Asp)	p.(Gly477Ser)	Likely pathogenic	Pathogenic	Dead at 5 years old, with profound intellectual and motor dissability
PA-40	c.676G > A	c.676G > A	p.(Gly226Arg)	p.(Gly226Arg)	Likely pathogenic	Likely pathogenic	Alive, 22 yaears old, with profound intellectual and motor dissability
PA-17	c.734C > A	c.1855C > T	p.(Ser245*)	p.(Arg619*)	Pathogenic	Pathogenic	Lost follow-up
PA-29	c.866_867delAG	c.1071G > T	p.(Glu289Valfs*53)	p.(Glu357Asp)	Pathogenic	Pathogenic	Dead at 1 year old, with neurodevelopmental impairment
PA-38	c.1071G > T	c.1071G > T	p.(Glu357Asp)	p.(Glu357Asp)	Pathogenic	Pathogenic	Alive, 2.3 years old, moderate intellectual and motor dissability
PA-24	c.1195C > T	NC_000013.11:g.(?_100089111)_(100089236_?)del (minimum deletion size: 126 bp) NC_000013.11(NM_000282.4):c.-10_105 + 11del	p.(Arg399Trp)	p.(?)	Pathogenic	Pathogenic	Dead at 15 years old, with profound intellectual and motor dissability
PA-30	c.1643 + 3 A > G	c.1643 + 3 A > G	p.(?)	p.(?)	VUS	VUS	Alive, 9.2 years old, moderate intellectual and motor dissability
PA-06	c.1899 + 4_1899 + 7delAGTA	c.1899 + 4_1899 + 7delAGTA	p.(?)	p.(?)	Pathogenic	Pathogenic	Alive, 10 years old, profound intellectual and motor dissability
PA-25	c.1899 + 4_1899 + 7delAGTA	c.2129_2130del	p.(?)	p.(Val710Alafs*23)	Pathogenic	Likely pathogenic	Dead at 3.3 years old, with profound intellectual and motor dissability
PA-34	NC_000013.11:g.(?_100089111)_(100089236_?)del (minimum deletion size: 126 bp) NC_000013.11(NM_000282.4):c.-10_105 + 11del	NC_000013.11:g.(?_100089111)_(100089236_?)del (minimum deletion size: 126 bp) NC_000013.11(NM_000282.4):c.-10_105 + 11del	p.(?)	p.(?)	Pathogenic	Pathogenic	Dead at 12.5 years old, with profound intellectual and motor dissability

PCCB							
Patient ID	Allele 1	Allele 2	Change protein 1	Change protein 2	ACMG classification allele 1	ACMG classification allele 2	Outcome
PA-18	c.484G > T	c.484G > T	p.(Gly162Trp)	p.(Gly162Trp)	Pathogenic	Pathogenic	Dead at 4.2 years old, with profound intellectual and motor dissability
PA-36	c.484G > T	c.484G > T	p.(Gly162Trp)	p.(Gly162Trp)	Pathogenic	Pathogenic	Alive, 4 years old, profound intellectual and motor dissability
PA-33	c. 1309G > A	c. 1309G > A	p.(Gly437Ser)	p.(Gly437Ser)	Pathogenic	Pathogenic	Alive, 8.6 years old, profound intellectual and motor dissability
PA-51	c. 1309G > A	c. 1309G > A	p.(Gly437Ser)	p.(Gly437Ser)	Pathogenic	Pathogenic	Alive, 1.9 years old, moderate intellectual and motor dissability
PA-32	c. 1309G > A	c.337C > T	p.(Gly437Ser)	p.(Arg113*)	Pathogenic	Pathogenic	Dead at 1 year old, with neurodevelopmental impairment
PA-43	c.1309G > A	c.517_518delTT	p.(Gly437Ser)	p.(Leu173Glyfs*56)	Pathogenic	Pathogenic	Dead at 3.4 years old, with profound intellectual and motor dissability
PA-15	c. 1309G > A	c.1388 T > G	p.(Gly437Ser)	p.(Met463Arg)	Pathogenic	Likely pathogenic	Dead at 3.2 years old, with profound intellectual and motor dissability
PA-22	c. 331C > T	c. 331C > T	p.(Arg111*)	p.(Arg111*)	Pathogenic	Pathogenic	Dead at 2.2 years old, with profound intellectual and motor dissability
PA-21	c.386_387delinsAAC	c.836C > T	p.(Phe129*)	p.(Pro279Leu)	Pathogenic	Pathogenic	Dead at 7 years old, with moderate intellectual and motor dissability
PA-07	c.484G > T	c.1173dupT	p.(Gly162Trp)	p.(Val392Cysfs*2)	Pathogenic	Pathogenic	Dead at 11 months old, with neurodevelopmental impairment
PA-01	c.1173dupT	c.1223delT	p.(Val392Cysfs*2)	p.(Ile408Thrfs*35)	Pathogenic	Pathogenic	Alive, 30 years old, moderate intellectual and motor dissability
PA-03	c.1218_1231delinsTAGAGCACAGGA	c.1218_1231delinsTAGAGCACAGGA	p.(Gly407Argfs*14)	p.(Gly407Argfs*15)	Pathogenic	Pathogenic	Alive, 3.4 years old, profound intellectual and motor dissability
PA-14	c.1218_1231delinsTAGAGCACAGGA	c.1218_1231delinsTAGAGCACAGGA	p.(Gly407Argfs*14)	p.(Gly407Argfs*15)	Pathogenic	Pathogenic	Dead at 3.5 years old, with profound intellectual and motor dissability
PA-35	c.1223delT	c.1231C > A	p.(Ile408Thrfs*35)	p.(His411Asn)	Pathogenic	Likely pathogenic	Dead at 3.5 years old, with profound intellectual and motor dissability
PA-42	c.1260dupT	c.1260dupT	p.(Glu421*)	p.(Glu421*)	Pathogenic	Pathogenic	Lost follow-up
PA-50	c.1173dupT	c.1173dupT	p.(Val392Cysfs*2)	p.(Val392Cysfs*2)	Pathogenic	Pathogenic	Alive, 5 months old, moderate intellectual and motor dissability

### 3.5 Genetic findings and phenotypic correlation

Thirty unrelated patients underwent molecular analysis: Of them, 46.66% (*N* = 14/30) carried clinically relevant variants in PCCA (7 homozygous), while the remaining 53.3% (*N* = 16/30) had a defect in *PCCB* (10 homozygous) (Table 1). Twenty-seven different variants were found, 14 in *PCCA* and 13 in *PCCB*. Four previously unreported variants were identified in *PCCA*, and one novel variant was found in *PCCB*. We did not find any VUS in *PCCB*, and only one VUS was found in *PCCA* [c.1643 + 3 A > G, or p.(?)] (Table 2). A comparison of genotypes constituted by LOF variants was performed between PCCA- and PCCB-related patients (Table S3). We found that 8 of 14 (57%) PCCA-related patients carried biallelic LOF variants, whereas in PCCB-related patients, 6 of 16 patients (37.5%) had biallelic LOF genotypes. There were no statistical differences between both groups.

**Table 2:** PCCA and PCCB variants in Mexican patients with propionic acidemia by frequency.

Allele	Change protein	Variant type allele	ACMG classification	“Previous reports dbSNP / LOVD Variant ID”	# alleles	% alleles
PCCA (NM_000282.4, *N* = 28 alleles)						
c.2041-1G > T	p.(?)	Splicing defect	Pathogenic (PVS1, PP5, PM2, PM3, PS4)	rs1367867218	5	17.9
c.1071G > T	p.(Glu357Asp)	Missense	Pathogenic (PM1, PM2, PP3, PP4, PS4)	rs761295534	4	14.3
**NC_000013.11:g.(?_100089111)_(100089236_?)del** **(minimum deletion size: 126 bp)** **NC_000013.11(NM_000282.4):c.-10_105 + 11del**	**p.(?)**	**In-frame and frameshift indels**	**Pathogenic (PVS1, PM2, PP4)**	**NPR / LOVD: #0001030178**	**3**	**10.7**
c.1899 + 4_1899 + 7delAGTA	p.(?)	In-frame and frameshift indels	Pathogenic (PS3, PM2, PM3, PP3, PP5)	rs794727334	3	10.7
**c.676G > A**	**p.(Gly226Arg)**	**Missense**	**Likely pathogenic (PP3, PP4, PM1, PM2)**	**NPR / LOVD: #0001030163**	**2**	**7.1**
c.866_867delAG	p.(Glu289Valfs*53)	In-frame and frameshift indels	Pathogenic (PVS1, PM2, PM3, PP5)	rs760976198	2	7.1
c.1643 + 3 A > G	p.(?)	Splicing defect	VUS (PP3, PM2)	rs2548307076	2	7.1
c.437 T > C	p.(Leu146Pro)	Missense	Likely pathogenic (PM1, PM2, PP3, PP4, PP5)	rs774457925	1	3.6
c.674G > A	p.(Gly225Asp)	Missense	Likely pathogenic (PP3, PM1, PM2, PM3)	rs2062161647	1	3.6
c.734C > A	p.(Ser245*)	Nonssense	Pathogenic (PVS1, PM2, PP5, PM3)	rs1594992191	1	3.6
c.1195C > T	p.(Arg399Trp)	Missense	Pathogenic (PM1, PM2, PM3, PM5, PP2, PP3, PP5)	rs1234167788	1	3.6
c.1429G > A	p.(Gly477Ser)	Missense	Pathogenic (PP3, PM1, PM2)	rs2548154575	1	3.6
c.1855C > T	p.(Arg619*)	Nonssense	Pathogenic (PVS1, PM2, PM3, PP5)	rs1194679272	1	3.6
c.2129_2130del	p.(Val710Alafs*23)	In-frame and frameshift indels	Likely pathogenic (PVS1, PM2, PM3, PP5)	rs1317003529	1	3.6

PCCB (NM_000532.5, *N* = 32 alleles)						
c.1309G > A	p.(Gly437Ser)	Missense	Pathogenic (PM1, PM2, PM3, PM5, PP2, PP3, PP5)	rs1349202366	7	21.9
c.484G > T	p.(Gly162Trp)	Missense	Pathogenic (PP3, PM1, PM2, PM3, PM5, PS4)	rs754752068	5	15.6
c.1173dupT	p.(Val392Cysfs*2)	In-frame and frameshift indels	Pathogenic (PVS1, PS3, PM2, PM3, PP5)	rs587776758	4	12.5
c.1218_1231delinsTAGAGCACAGGA	p.(Gly407Argfs*14)	In-frame and frameshift indels	Pathogenic (PVS1, PM2, PM3, PP5)	rs397507445	4	12.5
c.331C > T	p.(Arg111*)	Nonssense	Pathogenic (PVS1, PM2, PP5)	rs753981900	2	6.3
c.1223delT	p.(Ile408Thrfs*35)	In-frame and frameshift indels	Pathogenic (PVS1, PM2, PM3)	rs758309460	2	6.3
c.1260dupT	p.(Glu421*)	In-frame and frameshift indels	Pathogenic (PVS1, PM2, PM3, PP5)	rs1553784569	2	6.3
c.337C > T	p.(Arg113*)	Nonssense	Pathogenic (PVS1, PS3, PM2, PM3, PP5)	rs186031457	1	3.1
c.386_387delinsAAC	p.(Phe129*)	In-frame and frameshift indels	Pathogenic (PVS1, PM2, PM3, PP5)	rs398123463	1	3.1
c.517_518delTT	p.(Leu173Glyfs*56)	In-frame and frameshift indels	Pathogenic (PVS1, PM2, PM3, PP5)	rs755776820	1	3.1
c.836C > T	p.(Pro279Leu)	Missense	Pathogenic (PM2, PM3, PM5, PP2, PP3, PP5)	rs780837200	1	3.1
c.1231C > A	p.(His411Asn)	Missense	Likely pathogenic (PM1, PM2, PM3, PM5, PP3)	rs201751368	1	3.1
**c.1388 T > G**	**p.(Met463Arg)**	**Missense**	**Likely pathogenic (PP3, PM1, PM2, PM3)**	**NPR / LOVD: #0001030166**	**1**	**3.1**

New variants are shown in bold, NPR: not previously reported.

### 3.6 Genotypic *PCCA* spectrum

Missense variants were the most common pathogenic *PCCA* changes (42.9%) (Table 2). The splicing defect, c.2041-1G > T or p.(?) [rs1367867218, ClinVar ID: 648058], was the most frequent *PCCA* allele (*N* = 5/28 alleles, 17.9%), and it was identified in a homozygous state in two patients (Table 1, IDs: PA-13 and PA-28). This variant was classified as likely pathogenic in ClinVar, and our review of the patients' clinical and genotypic data led us to reclassify it as pathogenic, based on it meeting the PVS1, PS4, PM2, PM3, and PP5 criteria of ACMG/AMP [20] (Table 2). Both of the homozygous c.2041-1G > T patients exhibited severe phenotypes characterized by neonatal-onset symptoms requiring immediate hospitalization. The first case manifested at 4 h of life, with tachypnea, vomiting, and feeding refusal, followed by respiratory distress, lethargy, and limb clonus. The clinical condition progressively deteriorated, requiring mechanical ventilation. Biochemical findings included hyperammonemia, ketonuria, metabolic acidosis, leukopenia, and thrombocytopenia. The second patient developed symptoms on the second day of life, presenting vomiting, tachypnea, and respiratory distress, which required ventilatory support. Metabolic acidosis, ketonuria, and hyperammonemia were documented. Both patients died, one at the age of 1 month 6 days, and the other at 1 year 5 months. c.1071G > T or p.(Glu357Asp) was the second most common *PCCA* variant (*N* = 4/28 alleles, Table 2). One patient carried it in a homozygous state (Table 1, ID PA-38). Interestingly, she presented a less severe form of the disease; at the time this report was written, she was 2.3 years old and presented moderate intellectual and motor disabilities.

The first novel *PCCA* variant was the pathogenic NC_000013.11:g.(?_100089111)_(100089236_?)del or NC_000013.11(NM_000282.4):c.-10_105 + 11del variant, which leads to a 126-base pair deletion. In our cohort, this variant was found in the homozygous state in one patient (Table 1, Patient ID PA-34) and in the compound heterozygous state in another one (Table 1, Patient ID PA-24) in combination with the c. 1195C > T or p.(Arg399Trp) [rs1234167788; ClinVar ID: 662772] pathogenic variant. Both patients survived until adolescence and presented profound intellectual and motor disability; they died at 12.5 and 15 years old, respectively.

**Fig. 3:**
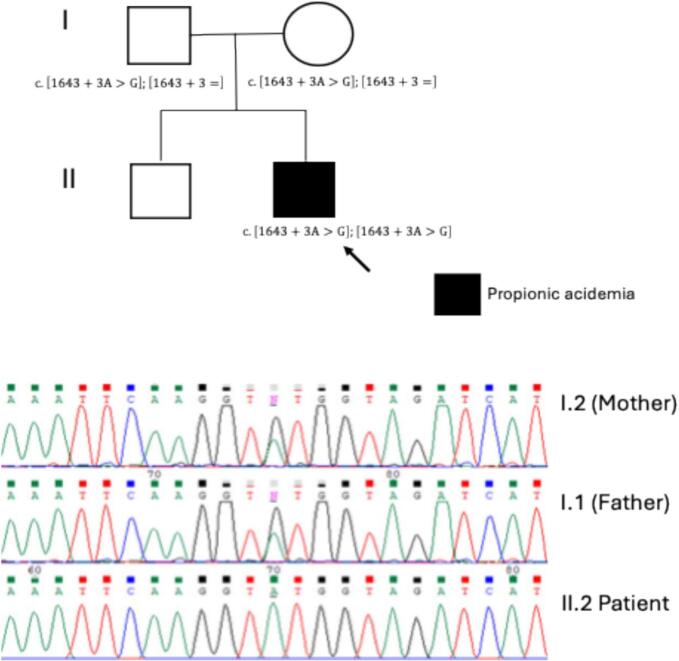
Genealogy of patient PA-30 and the identified *PCCA* genotype. The maternal and paternal origin of the c.1643 + 3 A > G or p.(?) variant was confirmed in II.2. The carrier status in both parents confirmed the homozygous c.[1643 + 3 A > G];[1643 + 3 A > G] genotype in II.2.

The second novel *PCCA* variant, c.676G > A or p.(Gly226Arg), was found in the homozygous state in one patient (Table 1, ID PA-40). Interesting, this was the only patient in whom PA was detected early by NBS. However, despite the prompt initiation of treatment, his biochemical and clinical outcomes were unsuccessful, and he had profound intellectual and motor disabilities. The third novel variant in *PCCA* was the splicing defect, c.1643 + 3 A > G or p.(?), which was classified as a VUS. This variant was found in a homozygous state in one patient (Table 1, ID PA-30), who still alive as of the writing of this report and had moderate intellectual and motor disability. To reclassify this variant, we performed segregation studies in both parents, confirming their carrier status (Fig. 3).

The fourth novel *PCCA* variant, nonsense variant c.734C > A or p.(Ser245*), was considered pathogenic by the ACMG/AMP criteria, and it was identified in a compound heterozygous patient (Table 1, ID PA-17). Unfortunately, this patient was lost to follow-up.

### 3.7 Genotypic *PCCB* spectrum

Of the *PCCB* pathogenic alleles, 46.1% were frameshift indels (Table 2). c.1309G > A or p.(Gly437Ser) [rs1349202366; ClinVar ID: 836321] was the most common clinically relevant *PCCB* variant (*N* = 7/32 alleles, 21.9%). It was found in a homozygous state in two patients (Table 1), who presented symptoms at 1 week (ID PA-33) and 6 months (ID PA-51) of age. Their symptoms comprised somnolence, feeding refusal, vomiting, respiratory distress, irritability, hypotony, hematological abnormalities, metabolic acidosis, and hyperammonemia. At the time of this writing, both patients were alive and under nutritional treatment but had intellectual and motor disability.

Other frequently observed *PCCB* variants were c.484G > T or p.(Gly162Trp) [*N* = 5/32 alleles, rs754752068; ClinVar ID: 529437] and c.1218_1231delinsTAGAGCACAGGA or p.(Gly407Argfs*14) [*N* = 4/32 alleles, rs397507445; ClinVar ID: 38875]. Homozygous genotypes were documented in two severely affected patients for each variant (Table 1, IDs PA-18, PA-36, PA03, and PA-14). These individuals exhibited early disease onset with profound intellectual and motor disabilities, and two of them died (ID: PA-18 and PA-14).

The likely pathogenic novel *PCCB* variant, c.1388 T > G or p.(Met463Arg), was found in a compound heterozygous state with the pathogenic c.1309G > A allele. This patient (Table 2, ID PA-15) presented the classical severe clinical picture and died at 3.2 years old with profound intellectual and motor disability.

### 3.8 Structural modeling of novel missense variants

**Fig. 4:**
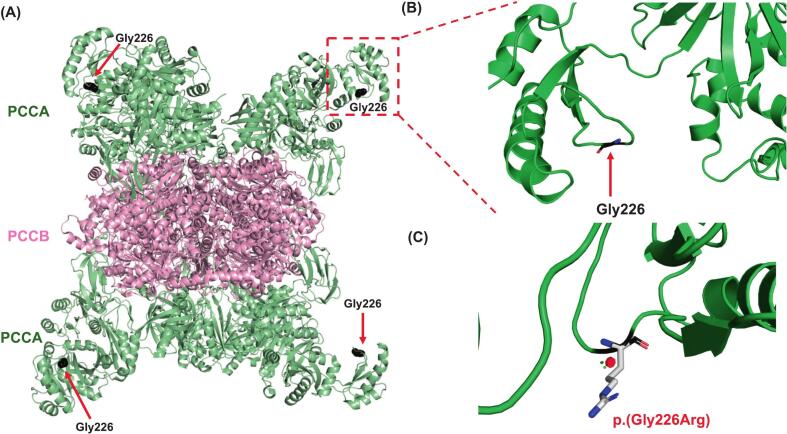
Crystallographic structure of the propionyl-CoA carboxylase protein complex and location of the Gly226 residue. (A) General overview of the protein complex comprising six subunits of PCCA layered on top and beneath (3 subunits per layer) the six subunits of PCCB. The location of Gly226 in PCCA is highlighted. (B) Zoomed view of the location of Gly226. (C) Steric clashes are provoked in the structure of PCCA when Gly226 is substituted with Arg.

The quaternary structure of the PCCA-PCCB complex is shown in Fig. 4A. This protein complex takes on a sandwich form, with six PCCA subunits layered on top of and below (3 per layer) the six PCCB subunits.

#### 3.8.1 *PCCA* p.(Gly226Arg)

This variant involves the Gly226 residue, which is located at the biotin carboxylase domain and forms part of a mobile beta hairpin. The SASA of the Gly226 residue is 60.7%, and its B′-factor is 1.94, indicating that a high proportion of this residue is exposed to the solvent area (Fig. 4B). The Arg substitution of Gly226 increased the SASA to 93.6% and decreased the B′-factor to 0, predicting even greater exposure of this residue to the solvent area (Fig. 4C). This increased exposure would be likely to provoke steric clashes with neighboring residues.

#### 3.8.2 *PCCB* p.(Met463Arg)

**Fig. 5:**
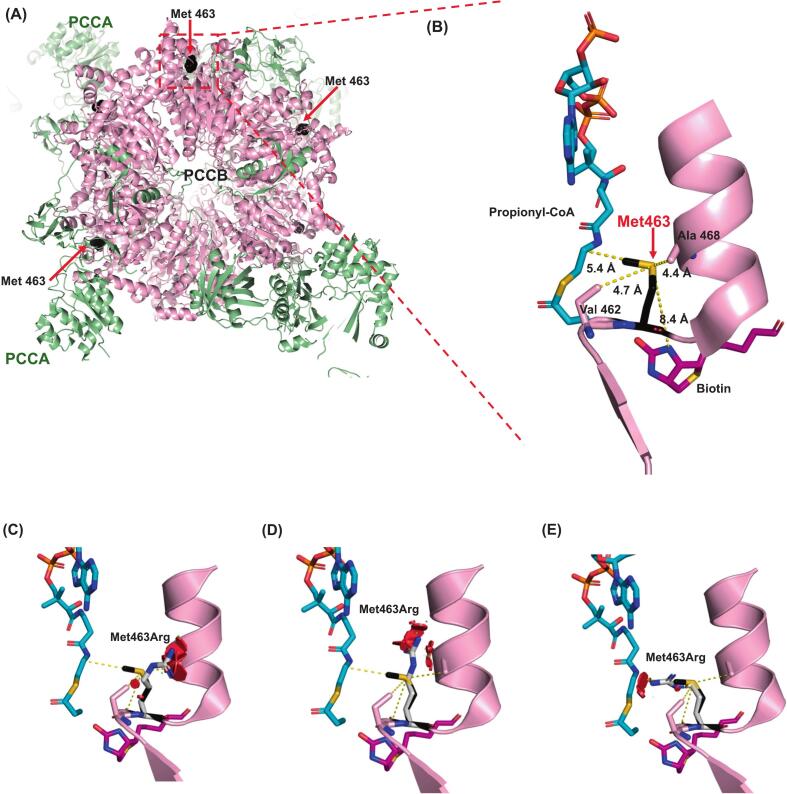
Crystallographic structure of propionyl-CoA carboxylase protein complex and location of the Met463 residue. (A) Location of Met463 in the overall structure of the PCCB complex. (B) Detailed location Met463, which is located near the catalytic domain. (C-E) Steric clashes within the neighborhood of Met463 are provoked when it is substituted by arginine.

This variant affects the C-terminal end of the carboxyl transferase domain of the PCCB protein (Fig. 5A). It has a SASA of 49% and a B′-factor of −0.16, indicating that it is not highly solvent-exposed. The sulfur atom of Met463 is in close contact with the substrate (5.4 Å) and the cofactor, biotin (8.4 Å) (Fig. 5B). The Arg substitution of this residue increased the SASA to 56.6% and decreased the B′-factor to −2.09, indicating that the residue's solvent exposure was greatly increased. This is likely to cause repulsive steric clashes with Ala468 and Val462 and to interfere with the substrate/biotin interaction (Fig. 5C-E).

## 4 Discussion

This study, which presents the largest cohort of Mexican patients with PA described to date, provides valuable insights into the clinical and genetic characteristics of this Latin American population. Our findings confirm the severe nature of PA, as evidenced by a high early mortality rate (66.7%), early symptom onset and a considerable diagnostic odyssey, with a mean time of 4.4 months from symptom onset to diagnosis. Clinical presentation was dominated by feeding difficulties, hyperammonemia, and metabolic acidosis, with symptom onset typically occurring within the first month of life. The neurological outcome in most patients was poor, and at the last follow-up, all the patients had neurological impairment. These observations are consistent with previous reports describing the severity of the disease[1], [29], [30].

High mortality rates and unfavorable outcomes have also been documented in other large patient cohorts [31], despite nutritional support and conventional pharmacological therapy, underscoring the persistent need for improved therapeutic strategies. The causes of death observed in this study (Table 3) were similar to those previously reported [31], [32].

**Table 3:** Immediate and contributing causes of death in patients with propionic acidemia in Mexico (*n* = 34). Causes were not mutually exclusive.

Category	Cause of death	Number of events (%)
Metabolic decompensation-related	Acute metabolic decompensation with metabolic acidosis	20 (58.8)
	Hyperammonemia crisis	15 (44.1)
Infection-related complications	Septic shock	12 (35.3)
	Pneumonia	7 (20.6)
	Fungal sepsis	2 (5.9)
	Peritonitis	1(2.9)
Organ failure and systemic complications	Respiratory failure	7 (20.6)
	Acute renal failure	7 (20.6)
	Cardiac failure	3 (8.8)
	Multiorgan failure	3 (8.8)
	Liver failure	2 (5.9)
	Bone marrow failure (pancytopenia with immunodeficiency)	2 (5.9)
	Pancreatitis	1 (2.9)
	Non-hyperammonemia encephalopathy	1 (2.9)
	Cerebral edema	1 (2.9)
	Cerebral hemorrhage	1 (2.9)
Cardiovascular complications	Cardiomyopathy	1 (2.9)
	Arrythmia	1 (2.9)
Other causes	Post-surgical gastrostomy complications	1 (2.9)

When stratified by genotype, patients with PCCB-related PA, showed a higher frequency of clinical manifestations, which was statistically different (Fig. 1). Going beyond this observation, we compared the proportion of LOF variants in the genotypes between PCCA- and PCCB-related patients; however, we did not find any statistical difference. Moreover, the comparison of the survival curves between both groups was not statistically different (Fig. 2). Thus, the findings of frequency of clinical manifestations should be interpreted with caution, as genotype-specific differences in severity have not been consistently demonstrated in previous studies. The observations in our cohort may therefore reflect sample size limitations, referral bias, or clinical heterogeneity rather than a true biological effect. Larger multicenter studies will be necessary to clarify whether these differences represent genuine genotype-phenotype correlations or population-specific factors.

Contextual healthcare factors may also contribute to the poor outcomes observed. Many patients must travel long distances to reach our center, limiting access to regular follow-up and optimal metabolic management [7]. This situation highlights the need to expand the availability of specialized metabolic centers in our country. In addition, access to ammonia-scavenging agents remains limited; sodium benzoate is the only medication consistently available. Recently, carglumic acid has become available at our institution, and to date, two patients have been treated with favorable outcomes. Furthermore, although pediatric liver transplantation programs have demonstrated feasibility in Mexico, their availability remains restricted to a small number of centers, and significant referral barriers persist [33]. Our institutional liver transplantation program has been active for approximately five years; however, none of the patients with PA included in this cohort have undergone transplantation, which likely contributes to the unfavorable outcomes observed and reflects ongoing barriers to accessing this therapeutic option.

### 4.1 Implications for NBS

Although prior reports questioned the clinical benefit of NBS for PA and methylmalonic acidemia [34], in part due to the early onset of symptoms in most patients, other studies have demonstrated that there is significant value in early detection through NBS. For instance, Chapman [9] and Baumgartner [10] emphasized that early diagnosis enables prompt metabolic stabilization, potentially reducing irreversible neurological damage and mortality. In our study, the high mortality rate strongly supports the idea that NBS should be implemented for PA, even when clinical manifestations arise within the first hours of life. This aligns with the principle that earlier diagnosis, while not always preventing symptom onset, can still meaningfully reduce diagnostic delays and improve survival outcomes.

In this cohort, only one patient (ID PA-40) was detected through newborn screening (NBS), yet the clinical outcome remained unfavorable despite early therapeutic intervention. This case illustrates that early diagnosis alone is insufficient to ensure optimal prognosis in PA when continuity of specialized care is not maintained. NBS was promptly conducted in the United States, allowing early diagnosis and timely treatment initiation; however, after returning to Mexico, the patient discontinued specialized metabolic follow-up. The subsequent loss of medical supervision was associated with severe metabolic decompensation and the development of irreversible neurological sequelae.

This case underscores the importance of sustained, lifelong access to specialized metabolic care, including ongoing biochemical monitoring and structured caregiver education, for individuals with PA. Moreover, it highlights the need for improved coordination of the healthcare system and robust long-term follow-up strategies, particularly in settings where access to metabolic expertise is limited.

An important added benefit of NBS for PA is its ability to facilitate genetic counseling, which is crucial for informed reproductive decision making. PA is an autosomal recessive disorder, and early identification and genotypic confirmation allow genetic counseling for parents, who are obligate heterozygous carriers, and thus face a recurrence risk of 25% in each subsequent pregnancy. Moreover, informed parents may consider utilizing preventive reproductive options, such as prenatal diagnosis or preimplantation genetic testing [35].

### 4.2 Genotypic findings and functional impact of variants

Among the 30 unrelated patients who underwent molecular analysis, a relatively even distribution of variants was observed between *PCCA* (46.7%) and *PCCB* (53.3%). A high level of allelic heterogeneity was observed, with 27 distinct variants identified, five of which were novel (Table 2). Interestingly, among the 28 *PCCA* alleles analyzed, the most frequent variant was the canonical splice site defect, c.2041-1G > T (17.85%). This variant is likely to abolish proper mRNA splicing, as often observed in severe, early-onset PA, where nonfunctional transcripts result in the absence of a functional enzyme [35]. Our two patients homozygous for this variant presented severe forms of the disease with profound neurological impairment; one of them died at 1.2 months of age, and the other at 1.4 years of age (Table 1). This early and fatal outcome for homozygous *PCCA* c.2041-1G > T patients was also seen in a previously reported Mexican infant with PA who died at 1 month of age, whose parents were heterozygous for this pathogenic splicing variant[35]. We cannot currently suggest a founder effect, as all patients carrying the c.2041-1G > T variant, as well as the parents of the previously reported infant[35], came from different regions of Mexico.

The second most common *PCCA* variant, c.1071G > T or p.(Glu357Asp), was found in the homozygous state in a single patient who presented a less severe form of the disease. This variant substitutes the wild-type amino acid with one that has similar physicochemical properties (a negatively charged group) and a similar molecular ratio. Therefore, it is reasonable that this change would not significantly impair the enzyme's structure, which is consistent with the patient's moderate phenotype. In the future, functional studies should seek to confirm this hypothesis. Importantly, the Glu357 residue acts as a base for the protonated bicarbonate, thereby playing a significant role in the catalytic process [36], [37].

We identified one patient with a compound heterozygous PCCA genotype comprising c.1195C > T, or p.(Arg399Trp), and a novel large intragenic deletion (ID: PA-24). This patient presented a very severe clinical picture: Her symptoms initiated at 7 days old, and she experienced multiple metabolic crises requiring hospitalization. She died at 15 years old with profound intellectual and motor disability. The Arg399 residue is crucial for the catalytic activity of the protein complex, as it stabilizes the intermediate and facilitates the formation of the carboxylated biotin [36], [37]. Substituting this residue with tryptophan is likely to impair catalysis, which could explain at least part of the severe clinical picture observed in this patient.

The novel *PCCA* missense variant, c.676G > A or p.(Gly226Arg), was found in the homozygous state in patient PA-40 (Table 1). In silico analysis predicted that repulsive clashes could occur when Gly is substituted by Arg in this context (Fig. 4C). These repulsive clashes could reflect the physicochemical differences in the nature of Arg, which carries a positive charge and is larger than Gly, and might trigger structural alterations not only at short distances but also at long distances. Moreover, the beta hairpin structural motif containing Gly226 is associated with mobile areas of the protein. The addition of positively charged Arg would be likely to compromise the mobility of the beta hairpin and provoke repulsive steric clashes in the neighborhood, especially in nearby structural motifs (Fig. 4C). Gly226 is also adjacent to a Lys residue, which has the same chemical nature as Arg. Having two positively charged residues in the beta hairpin, rather than one, would further impair its necessary mobility.

The only VUS variant found in *PCCA* was c.1643 + 3 A > G or p.(?), which meets ACMG/AMP criteria PM2, PP3, and PP4. In silico evaluation of this intronic variant, performed using the splicing prediction tool of Alamut Visual Plus version 1.13 (Alamut Visual Plus version v1.13 | © 2025 SOPHiA GENETICS), predicts only a discrete weakening of the canonical donor-acceptor splicing site of intron 18. Sanger sequencing confirmed the homozygous state in the patient (PA-30) and the heterozygous carrier status in both healthy parents (Fig. 3), consistent with the autosomal recessive inheritance pattern of propionic acidemia. However, this segregation evidence was not sufficient to reclassify the variant, which remains as a VUS according to ACMG/AMP guidelines. Further functional assays are warranted to assess the impact of this variant on splicing and determine its pathogenicity. Given that the patient exhibits a phenotype highly specific for PA, including metabolic acidosis, hyperammonemia, and elevated of biomarkers such as C3, 3-OH-propionic acid, and propionylglycine, future experimental validation could support an eventual reclassification toward likely pathogenic.

In *PCCB,* the most common variant was the missense alteration, c.1309G > A or p.(Gly437Ser). Gly437 forms an oxyanion hole that binds to the biotin carbonyl group. Therefore, substituting this key residue with serine would impact the catalytic process [37], [38]. This variant was first described in the Turkish population [39]. Later, Kraus identified the same variant in another Turkish patient [40]. Here, we found the variant in a homozygous state in two patients (IDs: PA-33 and PA-51). Remarkably, both patients came from Jalisco State, from a region known as *Los Altos de Jalisco*, where founder effects for other metabolic disorders, such phenylketonuria [41], have been noted. Therefore, further investigations are necessary to confirm a possible founder effect.

Our in silico analyses for the novel *PCCB* variant, c.1388 T > G, or p.(Met463Arg) (Fig. 5), which were performed using the crystallographic structure of the protein, predicted that this variant would cause repulsive clashes with the substrate and the cofactor, biotin. This might reflect the differences in the chemical natures of Met and Arg. Met provides flexibility to the helix in which it is located, reflecting the particular properties of its sulphuretted side chain [42]. In contrast, Arg residues form hydrogen bonds and electrostatic interactions, thereby increasing protein stability [43]. Thus, the absence of Met could decrease the flexibility of the helix, and the presence of Arg could decrease the electrostatic interactions with the substrate and cofactor, as well as their affinity for the protein. This novel variant was found in only one patient, in a compound heterozygous state (Table 1, PA-15); at present, the changes predicted by in silico analysis account for only part of the patient's severe clinical picture. Further functional studies are needed to correlate the in silico predictions with the resulting clinical picture.

### 4.3 Limitations

A significant proportion of patients lacked access to genetic testing (*N* = 21/51, 41%), and long-term follow-up data were incomplete for 13.7% of the cohort. Additionally, given that our institution functions as a tertiary referral center, this study may be affected by referral bias, as patients admitted to our facility are more likely to represent more severe cases or individuals who received initial management at other institutions. Also, the results of our in silico analyses, while informative, should be functionally validated through enzymatic and/or cellular assays to confirm the predicted pathogenicity of novel variants.

## 5 Conclusion

Here, in the first study describing a large cohort of PA patients in Mexico, we provide detailed clinical and genotypic characterizations. The most common variants identified in the *PCCA* and *PCCB* genes were c.2041-1G > T or p.(?) (17.9% of alleles) and c.1309G > A or p.(Gly437Ser) (21.9% of alleles), respectively. The predominance of early neonatal onset, severe neurological sequelae, and high mortality rate (66.7%), along with the notable proportion of patients with previously deceased siblings, highlights the need for decision makers to include PA in the expanded Mexican NBS program by incorporating C3 analysis. Additional necessary steps would include implementing standardized diagnostic protocols to minimize diagnostic delays and expanding access to genetic testing and counseling. Until PA screening is routinely performed at birth in Mexico, efforts are needed to increase pediatricians' awareness of the clinical picture, to support early detection and prompt management.

## Authors' contribution

**Marcela Vela-Amieva.** Designed the study, analyzed and curated data results, wrote the original draft, wrote, reviewed and edited the final version of the manuscript, and acquired funding.

**Miguel Ángel Alcántara-Ortigoza**. Performed variant interpretation, wrote the original draft, reviewed and edited the final version of the manuscript.

**Sara Guillén-López**. Provided nutritional care to the patients and reviewed and edited the final version of the manuscript.

**Lizbeth Alejandra López-Mejía**. Provided nutritional care to the patients, reviewed and edited the final version of the manuscript.

**Ariadna González-del Ángel**. Provided genetic counseling to patients and their families, performed variant interpretation, reviewed and edited the final version of the manuscript.

**Miriam Erandi Reyna-Fabián.** Isolated DNA from blood samples, performed variant interpretation, and reviewed and edited the final version of the manuscript.

**Liliana Fernández-Hernández**. Provided genetic counseling to patients and their families, performed variant interpretation, reviewed and edited the final version of the manuscript.

**Gabriel López-Velázquez**. Performed statistical analyses, reviewed and edited the final version of the manuscript.

**Daniela Mancera-Hernández**. Analyzed patients clinical records, curated the database, reviewed and edited the final version of the manuscript.

**Sergio Enríquez-Flores**. Performed in silico structural analysis of the protein variants, reviewed and edited the final version of the manuscript.

**Isabel Ibarra-González.** Analyzed biochemical data results and reviewed the final version of the manuscript.

**Cynthia Fernández-Lainez**. Designed the study, analyzed and curated data results, performed in silico protein modeling, wrote the original draft, wrote, reviewed and edited the final version of the manuscript, supervised the project and acquired funding.

## CRediT authorship contribution statement

**M. Vela-Amieva:** Writing – review & editing, Writing – original draft, Funding acquisition, Formal analysis, Data curation, Conceptualization. **M.A. Alcántara-Ortigoza:** Writing – review & editing, Writing – original draft, Methodology, Formal analysis. **S. Guillén-López:** Writing – review & editing, Methodology. **L. López-Mejía:** Writing – review & editing, Methodology. **A. González-del Ángel:** Writing – review & editing, Methodology, Investigation, Formal analysis. **M.E. Reyna-Fabián:** Writing - review & editing, Methodology, Investigation, Data curation. **L. Fernández-Hernández:** Writing – review & editing, Methodology, Investigation, Data curation. **Gabriel López-Velázquez:** Formal analysis, Writing – review & editing. **D. Mancera-Hernández:** Writing – review & editing, Methodology, Investigation, Data curation. **S. Enríquez-Flores:** Writing – review & editing, Visualization, Software, Methodology, Investigation. **I. Ibarra-González:** Writing – review & editing, Methodology. **C. Fernández-Lainez:** Conceptualization, Formal analysis, Data curation, Methodology, Writing - original draft, Writing – review & editing, Funding acquisiton, Project Supervision.

## Consent for publication

Written informed consent was obtained from the patients involved in the study or by their parents for the publication of this paper.

## Ethics approval and consent to participate

This study was conducted according to the guidelines of the Declaration of Helsinki and approved by the Institutional Review Board (Ethics, Research, and Biosafety Committees) of the National Institute of Pediatrics (Reference protocol numbers 2022/051 and 2024/032).

## Ethical considerations

The relevant institutional committees approved this study for research, ethics, and biosafety (protocol ID: 2024/032). Written informed consent for exome sequencing-based genotyping of *PCCA* and *PCCB* was obtained from the patients' legal guardians or parents. All participants underwent genetic counseling before and after genotyping.

## Funding

This research was funded by the Instituto Nacional de Pediatría, Secretaría de Salud (Recursos Fiscales 2022–2024, Programa E022 Investigación y Desarrollo Tecnológico en Salud, Ciudad de México, México, protocol numbers 2022/051 and 2024/032).

## Declaration of competing interest

None.

## Data Availability

Data will be made available on request.

## Appendix A Supplementary data

Supplementary material: Supplementary Table S3. Functional classification of variants found in Mexican patients with propionic acidemia.
